# Evaluation of antimalarial activity of leaves of *Acokanthera schimperi* and *Croton macrostachyus* against *Plasmodium berghei* in Swiss albino mice

**DOI:** 10.1186/1472-6882-14-314

**Published:** 2014-08-26

**Authors:** Tigist Mohammed, Berhanu Erko, Mirutse Giday

**Affiliations:** Hosanna Health Science College, Hosanna, P.O. Box 159, SNNPR, Ethiopia; Aklilu Lemma Institute of Pathobiology, Addis Ababa University, P.O. Box 1176, Addis Ababa, Ethiopia

**Keywords:** *Croton macrostachyus*, *Acokanthera schimperi*, Antimalarials, *Plasmodium berghei*, *in vivo*, Ethiopia

## Abstract

**Background:**

Malaria is one of the most important tropical diseases and the greatest cause of hospitalization and death. Recurring problems of drug resistance are reinforcing the need for finding new antimalarial drugs. In this respect, natural plant products are the main sources of biologically active compounds and have potential for the development of novel antimalarial drugs. A study was conducted to evaluate extracts of the leaves of *Croton macrostachyus* and *Acokanthera schimperi* for their *in vivo* antimalarial activity.

**Methods:**

The plants were selected based on their ethnomedicinal information. Acute and sub- acute toxicity studies of the crude extracts were carried out in Swiss albino mice. To assess the effect of extracts of the plants on the parasite, a 4-day suppressive standard test was performed using *Plasmodium berghei* (ANKA strain). Data were analyzed using paired t-test and ANOVA.

**Results:**

In acute toxicity study, the two plants extracts did not show any sign of toxicity up to 2000 mg/kg. In sub-acute toxicity study, both plants did not exhibit any hematological change and mortality throughout the observation period up to the highest dose of 1000 mg/kg given daily. Extracts of the leaves of both plants significantly (P < 0.05) suppressed parasitaemia in dose dependent manner at all dose levels.

**Conclusions:**

The findings may support the traditional use of the plants to treat malaria. Further pharmacological, toxicological and phytochemical studies are, however, required to evaluate the potential of the plants towards the development of new antimalarial agent.

## Background

Malaria is a major public-health problem in the world; transmission primarily being common in tropical and subtropical regions [1]. According to WHO, globally, there were about 216 million and 207 million cases of malaria in 2010 and 2012, respectively, and estimated 655,000 and 627,000 deaths in 2010 and 2012, respectively [2, 3].

In Ethiopia, though some improvements were recently achieved, malaria is still the leading cause of morbidity and mortality [4, 5]. Ethiopia is also one of the most malaria epidemic-prone countries in Africa where rates of morbidity and mortality increase 3–5 folds during epidemics [6].

Multi-drug resistant strains of the parasite to antimalarial drugs proved to be a challenging problem in malaria control in most parts of the world [1]. These recurring problems render development and promotion of phytomedicines as alternative solution to malaria control [7]. Medicinal plants have been playing a vital role in the treatment of malaria for centuries [8] and have always been considered to be a possible alternative and rich source of new drugs. Today, herbal products are being used worldwide in a variety of healthcare settings, and as home remedies [9]. Over 1200 plant species from 160 families are used to treat malaria and fever in endemic countries [10].

It is estimated that about 80% of the population in Ethiopia is still dependent on traditional medicines [11]. However, scientific studies on the status of use of phytomedicine, preparation of crude extracts and isolation of active principles is very minimal [12]. *Croton macrostachyus* and *Acokanthera shimperi* are two of the plants that are traditionally used for malarial treatment in Ethiopia [13, 14]. A study reported that methanol extracts of the fruits of *C. macrostachyus* have shown substantial antimalarial activities against *P. falciparum in vitro* with IC_50_ value of 0.94 μgm/mL [15]. An *in vivo* study also showed that methanol extract of the fruits of *Croton macrostachyus* has significant suppressive effect against *P. berghei* [16]. An *in vivo* study conducted on *Acokanthera oppositifolia* in Kenya and *in vitro* study conducted on *Croton zambesicus* in Cameroon, taxonomically related species to *A. schimperi* and *C. macrostachyus*, respectively, demonstrated an interesting antiplasmodial activity on *P. falciparum* [17, 18]. The objective of this study, therefore, was to investigate antimalarial effect of aqueous and methanol extracts of the leaves of *A. schimperi* and *C. macrostachyus* in mice infected with chloroquine sensitive strain of *P. berghei*. The study was conducted at the Endod and Other Medicinal Plants Research Unit of the Aklilu Lemma Institute of Pathobiology, Addis Ababa University.

## Methods

### Plant material collection sites

Young leaves of *Acokanthera schimperi* were collected from Bishan Gari Lodge area (Buku Wolda) about 20 km off the Addis Ababa – Hawasa road in December 2012 (reference number TM02-2012) and that of *Croton macrostachyus* from the compound of the Aklilu Lemma Institute of Pathobiology (ALIPB) in October, 2012 (reference number: TM01-2012). The plants were identified by the second author, the botanist at the Aklilu Lemma Institute of Pathobiology, Addis Ababa University, and voucher specimens were deposited at the Institute. The plants were selected based on previous reports of their traditional use for treating malaria in Ethiopia [13, 14].

### Preparation of crude plant extracts

The leaves of *Acokanthera schimperi* and *Croton macrostachyus* were washed thoroughly with running tap water and each plant material was reduced to small fragments. The plant samples were air-dried at room temperature under shade, and ground into powder using pestle and mortar. The powdered plant materials were packed in plastic bags until extraction. The coarsely powdered plant materials were weighed using sensitive balance and repeatedly extracted in water and methanol in maceration flasks. Powdered plant parts (140 gm of *Croton macrostachyus* and 155 gm of *Acokanthera schimperi*) were mixed in distilled water and methanol separately in 1:10 (w/v) and placed on orbital shaker at 130 rpm for 24 hours in water and for 72 hours in methanol at room temperature. Then, the extracts, were filtered through Whatman (no 1, diameter 150 mm, England). The methanol extract was concentrated at 40°C with a rotary evaporator (Buchi, Switzerland) in distillation flask to eliminate methanol from the crude extract and concentrated further to dryness in a water bath at 40°C. For aqueous extract, residual water was removed by lyophilizer (Heto Power Dry LL3000, Wagtech, Denmark) at -56°C. Then, the extracts were stored in tightly closed bottle containers in a refrigerator at 4°C until they were used for the experiment [19–21].

### Experimental animals and parasite inoculation

#### Experimental animals and parasite strain

Male Swiss albino mice, 5–7 weeks of age and 23–38 gm of weight were obtained from the Ethiopia Health and Nutrition Research Institute (EHNRI). They were kept in the animal care facility of the Aklilu Lemma Institute Pathology, Addis Ababa University, for one week prior to the initiation of the experiments for acclimatization to laboratory conditions. The mice were kept at room temperature in humid environment and were exposed to 12 hours light and 12 hours darkness. The mice were given water and standard food pellets every 24 hours. *In vivo* antimalarial testing in mice was done using chloroquine sensitive strain of *Plasmodium berghei* (ANKA strain) obtained from EHNRI. On weekly basis, the parasites were maintained by serial passage of blood from infected mice having a parasitamia level of 20-30% to non-infected ones.

#### Parasite inoculation

Albino mice previously infected with *P. berghei* were used as donor animals. The parasitaemia of the donor mice was first determined and parasitized erythrocytes were obtained by cardiac puncture using ethyl ether as anesthesia and diluted in physiological saline (0.9%). The dilution was made based on the parasitaemia of the donor mice and the RBC count of normal mice in such a way that 1 mL blood contains 5 × 10^7^ infected erythrocytes [22]. Each mouse was inoculated by intraperitoneal injection with a blood suspension (0.2 mL) containing 1 × 10^7^ parasitized erythrocytes.

### *In vivo*toxicity test of the crude plant extracts

The crude methanol and aqueous extracts of *Croton macrostachyus* and *Acokanthera schimperi* intended for the antimalarial test against *P. berghei* were evaluated for their acute toxicity in non-infected Swiss albino mice aged 6–8 weeks and weighing 23-38 g. For each extract, 10 mice were used by randomly dividing them into two groups of 5 mice per group. The mice were starved 3–4 hours before the experiment began with only water allowed and 1–2 hours after the extract was given [23]. Then, the mice in group 1 were given orally 0.2 mL of 2000 mg/kg body weight of the extract in single dose in dH2O. The mice in the control groups received 0.2 mL of vehicle of the extract (dH2O). Then, the mice were observed continuously for 30 minutes, followed by 4 hourly observation for 24 hours and once a day for the next 13 days, for any manifestation of toxicity [23].

For sub-acute toxicity studies, weight and haematological parameters were determined before and after treatment. These include, packed cell volume (PCV), white blood cells count (WBC) and haemoglobin (Hb). A hand-held scale reader for PCV, Hemacytometer (Hawksley improved neubauer double cell) for WBC and haemoglobin meter (HemoCue model 201^+^, Angelholm, Sweden) for Hb were used for the determination. The extracts in each case were administered orally for four days (i.e. D0 to D4) using gavages [23]. The mice were grouped randomly, five mice per group. The mice in group I, II and III were given orally 500, 750 and 1000 mg/kg body weight in single dose volume of 0.2 mL of each extract, respectively and control group received 0.2 mL of dH2O. The data was recorded on day 0 and day 4 (12 hours after administration of the last dose) with regards to reduction in PCV and Hb, weight losses and WBC, following which, the mice were closely observed for one month.

### *In vivo*antimalarial screening

Schizontocidal activity was evaluated by the 4-day suppressive standard test [24]. Male Swiss albino mice weighing 24–35 grams were randomly divided into five groups of five mice per cage for each extract. All mice were infected with *P. berghei* on the first day (D0). Treatment commenced 3 hours after infection on day 0 and then continued daily for four days (i.e. from day 0 to day 3). The mice in the three treatment groups received extract from the *Acokanthera schimperi* and *Croton macrostachyus* 0.2 mL (200 mg/kg, 400 mg/kg, and 600 mg/kg) of each extract. Group IV (negative control) received the vehicle (dH_2_O), while the fifth group received the standard antimalarial drug (chloroquine) 25 mg/kg, with the same amount of volume (0.2 mL), daily for 4 days. The plant extracts and the standard drug were administered through intragastric route using standard tube to ensure safe ingestion of the extracts and the drug [23]. On the fifth day (D4), blood samples were collected from tail snip of each mouse [25] and thin smears were prepared and stained with 10% Giemsa solution. Then, the slides were examined under the microscope and parasitaemia determined by counting minimum of five fields per slide (500 RBC), for negative smear up to 100 field [26]. The smears were counted blindly by technician. Percent parasitaemia and percentage of suppression was calculated using the formula indicated in [19, 27].


In acute toxicity testing, change in body weight of individual mice was determined and recorded before and after extract administration [23], while evaluation of antimalarial activity and sub toxicity testing, body weight of each mouse was measured before infection (day 0) and continuously for four days.

Packed cell volume measurement was done before infection on day 0 and on day 4. Blood was collected from tail of each mouse in heparinized microhaematocrit capillary tubes filled up to 3/4th of their length. Then, the blood was centrifuged using microhematocrit centrifuge at 12,000 rpm for 5 minutes [28] and measured using a hand scale reader.

In addition, mortality of mice was monitored daily and the number of days was recorded for each mouse in the treatment and control groups throughout the follow up period.

### Data analysis

Data were analyzed using computer software SPSS, version 16. Results of the study were expressed as a mean ± standard error of the mean (M ± SEM). Statistical significance was determined by one way analysis of variance (ANOVA) with multiple comparison tests (Post Hoc/Tukey’s test/HSD) to compare parameters within groups. Two tailed paired t-test was used to compare mean PCV, Hb, WBC and body weight before and after treatment. All data were analyzed at a 95% confidence interval (α = 0.05).

### Ethical consideration

The study obtained ethical clearance from Institutional Review Board (IRB) of Aklilu Lemma Institute of Photobiology, Addis Ababa University. The mice were handled in humane accordance to the National Guidelines for handling laboratory animals for its scientific and academic merits.

## Results

### Extraction yield of plants

The methanol and aqueous extracts of *Acokanthera schimperi* showed the highest yield with 37.4% and 23.4%, followed by methanol extract of *Croton macrostachyus* with 17.42% and that of water extract 4.6%.

### Acute toxicity test

In the *in vivo* acute toxicity studies of the plant extracts, there were no gross physical and behavioral changes; including, rigidity, sleep, diarrhea, depression, abnormal secretion and hair erection for 24 hours and no mortality occurred within the observation period of two weeks.

### Sub-acute toxicity test

Methanol and aqueous extracts of *Croton macrostachyus* on day 4 showed no statistically significant difference (P > 0.05) in all the hematological parameters mentioned above as compared to that of day 0 (Table 1). No mortality was observed in all treatment groups of both extracts and there was no statistical significant difference (P > 0.05) on body weight in methanol extract. Though, significant (P < 0.05) bodyweight loss at the highest dose i.e. 1000 mg/kg (from 30.20 gm to 25.90 gm) and body weight gain at 500 mg/kg (from 31.34gm to 33.58 gm) in aqueous extract and in negative control group (from 26.54 gm to 29.36 gm) was observed.

**Table 1:** **Sub-acute toxicity effects of methanol and aqueous extracts of** ***Croton macrostachyus***

Extract	Dose	Parameters	Day 0	Day 4	P-Value
**Methanol**	500 mg	Body weight(gm)	33.00 ± 2.59	32.34 ± 2.91	0.628
		PCV (%)	54.60 ± 1.29	54.20 ± 1.62	0.840
		WBC (mm^3^)	7360 ± 1591.34	10300 ± 250.49	0.114
		Hb	not determined	not determined	
	750 mg	Body weight (gm)	34.66 ± 2.29	35.92 ± 2.13	0.085
		PCV (%)	51.60 ± 0.93	50.20 ± 0.66	0.404
		WBC (mm^3^)	5180.00 ± 908.93	7470.00 ± 314.88	0.720
		Hb	not determined	not determined	
	1000 mg	Body weight(gm)	36.56 ± 1.10	33.14 ± 1.76	0.124
		PCV (%)	52.20 ± 1.96	52.20 ± 0.86	0.852
		WBC (mm^3^)	9420.00 ± 489.03	9840.00 ± 1259.41	0.647
		Hb	not determined	not determined	
	NC	Body weight (gm)	35.86 ± 0.56	40.12 ± 0.97	0.007
		PCV (%)	52.00 ± 0.89	52.40 ± 0.60	0.587
		WBC (mm^3^)	9700 ± 921.95	5500 ± 916.79	0.004
		Hb	not determined	not determined	
**Aqueous**	500 mg	Body weight (gm)	31.34 ± 0.56	33.58 ± 0.72	0.002
		PCV (%)	55.4 ± 1.50	50.80 ± 2.33	0.079
		WBC (mm^3^)	5530.00 ± 501.65	8870.00 ± 1750.90	0.152
		Hb	not determined	not determined	
	750 mg	Body weight (gm)	31.94 ± 1.09	29.12 ± 2.02	0.072
		PCV (%)	55.00 ± 1.18	53.60 ± 0 .87	0.245
		WBC (mm^3^)	9760.0 ± 932.12	13610 ± 1581.41	0.72
		Hb	not determined	not determined	
	1000 mg	Body weight (gm)	30.20 ± 1.49	25.90 ± 2.38	0.018
		PCV (%)	55.40 ± 2.14	57.60 ± 1.12	0.282
		WBC (mm^3^)	4700.00 ± 470.11	9730.00 ± 1503.46	0.53
		Hb	not determined	not determined	
	NC	Body weight (gm)	26.54 ± 1.16	29.36 ± 1.52	0.021
		PCV (%)	54.60 ± 0.75	47.40 ± 0.81	0.007
		WBC (mm^3^)	9630 ± 1260.22	8000 ± 1832.01	0.54
		Hb	not determined	not determined	

The results are expressed as mean ± SEM (n = 5); NC = negative control; PCV = packed cell volume: WBC = white blood cells; Hb = haemoglobin; P > 0.05 is not significant.

In sub-acute toxicity studies of methanol and water extracts of *A. schimperi*, when day 4 was compared to day 0 at a given dose levels (500,750,1000 mg/kg), no mortality was observed in any treatment group (Table 2). There was no significant difference (P > 0.05) observed on hematological parameters (PCV and Hb) and in the body weight of animals treated with both extracts and control group.

**Table 2:** **Sub-acute toxicity effects of methanol and aqueous extracts of** ***Acokanthera schimperi***

Extract	Dose	Parameters	Day 0	Day 4	P-Value
**Methanol**	500 mg	Body weight (gm)	31.74 ± 2.16	33.76 ± 2.08	0.162
		PCV (%)	54.60 ± 0.75	52.80 ± 1.50	0.195
		WBC	not determined	not determined	
		Hb (g/dL)	16.36 ± 0.55	15.38 ± 0.43	0.052
	750 mg	Body weight (gm)	30.38 ± 0.59	31.68 ± 0.84	0.094
		PCV (%)	55.40 ± 0.40	50.20 ± 2.18	0.057
		WBC	not determined	not determined	
		Hb (g/dL)	17.10 ± 0.32	16.32 ± 0.63	0.074
	1000 mg	Body weight (gm)	32.22 ± .1.54	31.70 ± 1.72	0.073
		PCV (%)	52.40 ± 0.75	49.80 ± 2.04	0.395
		WBC	not determined	not determined	
		Hb (g/dL)	16.46 ± 0.27	14.68 ± 0.63	0.109
	NC	Body weight (gm)	29.04 ± 1.82	30.80 ± 1.59	0.078
		PCV (%)	52.40 ± 0.98	51.20 ± 1.07	0.109
		WBC	not determined	not determined	
		Hb (g/dL)	16.58 ± 0.79	15.48 ± 0.65	0.097
**Aqueous**	500 mg	Body weight (gm)	30.42 ± 1.45	31.52 ± 1.30	0.327
		PCV (%)	53.30 ± 1.41	50.20 ± 0.66	0.156
		WBC	not determined	not determined	
		Hb (g/dL)	16.66 ± 0.38	14.86 ± 0.35	0.052
	750 mg	Body weight (gm)	31.34 ± 2.34	31.88 ± 2.11	0.573
		PCV (%)	53.10 ± 1.33	49.40 ± 1.25	0.083
		WBC	not determined	not determined	
		Hb (g/dL)	16.30 ± 0.72	14.96 ± 0.77	0.123
	1000 mg	Body weight (gm)	31.60 ± 2.36	32.90 ± 2.21	0.147
		PCV (%)	54.48 ± 0.96	51.20 ± 2.15	0.227
		WBC	not determined	not determined	
		Hb (g/dL)	17.10 ± 0.51	15.54 ± 0.43	0.123
	NC	Body weight (gm)	29.04 ± 1.82	30.80 ± 1.59	0.078
		WBC	not determined	not determined	
		PCV (%)	52.40 ± 0.98	51.20 ± 1.07	0.109
		Hb (g/dL)	16.58 ± 0.79	15.48 ± 0.65	0.097

The results are expressed as mean ± SEM (n = 5); NC = negative control; PCV = packed cell volume; WBC = white blood cells; Hb = haemoglobin; P > 0.05 is not significant.

### Antimalarial activities

#### Effect of crude extracts on PCV and body weight

Methanol and aqueous extracts of *Croton macrostachyus* did not prevent PCV reduction due to parasitemia. Significant reduction of PCV on day 4 as compared to day 0 (p < 0.05) was observed in both treatment and in negative control groups. But, no significant (p > 0.05) change was observed in body weight of all groups. For chloroquine treatment groups, no significant change (P > 0.05) was observed in all parameters (Table 3).

**Table 3:** **Effect of crude methanol and aqueous extracts of** ***Croton macrostachyus*** **on body weight and PCV of** ***P*** **.** ***berghei*** **infected mice**

Extract/Treatments	Doses (mg/kg)	PCV		Body weight in grams	
		Day0	Day4	Day0	Day4
**Methanol extracts**	200	54.60 ± 0.51	45.60 ± 1.17 ^a^	30.06 ± 0.50	30.06 ± 0.61*
	400	55.70 ± 0.30	46.00 ± 2.98 ^a^	29.68 ± 0.51	28.68 ± 0.10*
	600	54.68 ± 0.63	46.00 ± 1.30 ^a^	30.60 ± 1.55	30.04 ± 1.43*
**Distilled water**	1 mL	55.00 ± 0.84	44.00 ± 1.80 ^a^	28.78 ± 1.10	28.18 ± 1.07*
**Chloroquine**	25	53.20 ± 1.59	49.00 ± 1.09^*^	29.96 ± 0.62	29.80 ± 0.66^*^
**Aqueous extracts**	200	55.60 ± 0.24	43.20 ± 3.18 ^a^	30.20 ± 1.75	28.18 ± 1.07*
	400	55.60 ± 0.24	45.60 ± 1.36 ^a^	28.32 ± 1.60	28.66 ± 2.26*
	600	55.60 ± 0.24	45.00 ± 1.92 ^a^	29.00 ± 0.95	28.48 ± 1.65*
**Distilled water**	1 mL	55.00 ± 0.84	44.00 ± 1.80 ^a^	28.78 ± 1.10	28.18 ± 1.07*
**Chloroquine**	25	53.20 ± 1.59	49.00 ± 1.09*	29.96 ± 0.62	29.80 ± 0.66 ^*^

Results presented as mean ± SD; n = 5; * = there was no significant difference, between Day-0 and Day-4 (P > 0.05); a = there was a significant difference between Day-0 and Day-4 (P < 0.05).

PCV and body weight measurements on day 4 indicated that both methanol and aqueous extracts of *Acokanthera schimperi* prevented significantly (P < 0.05) PCV reduction and body weight loss at all dose levels (200 mg, 400 mg, 600 mg) due to parasitemia. In negative control, there was significant (P < 0.05) reduction in PCV on day 4, though no bodyweight change observed (Table 4).

**Table 4:** **Effect of crude methanol and aqueous leaf extracts of** ***Acokanthera schipmeri*** **on body weight and PCV of** ***P*** **.** ***berghei*** **infected mice**

Extracts/Treatments	Dose (mg/kg)	PCV		Body weight in grams	
		Day 0	Day 4	Day 0	Day 4
**Methanol extracts**	200	55.80 ± 0.37	49.20 ± 2.71*	29.20 ± 1.22	29.08 ± 1.09*
	400	53.60 ± 0.40	52.80 ± 1.50*	31.82 ± 2.036	31.34 ± 2.34*
	600	55.20 ± 0.37	46.20 ± 3.39*	29.98 ± 1.63	29.88 ± 0.89*
**Distilled water**	1 mL	55.20 ± 1.11	37.00 ± 2.94^a^	29.64 ± 1.99	26.96 ± 2.26*
**Chloroquine**	25	54.00 ± 1.14	55.60 ± 1.50*	28.20 ± 1.79	28.68 ± 2.10*
**Aqueous extracts**	200	53.60 ± 1.50	49.20 ± 1.80*	28.80 ± 1.53	28.90 ± 1.55*
	400	54.00 ± 1.14	48.60 ± 1.69*	29.78 ± 2.08	28.14 ± 1.61*
	600	52.20 ± 1.24	45.20 ± 2.92*	30.24 ± 1.48	28.52 ± 1.48*
**Distilled water**	1 mL	55.20 ± 1.11	37.00 ± 2.94^a^	29.72 ± 1.95	26.90 ± 2.28*
**Chloroquine**	25	54.00 ± 1.14	55.60 ± 1.50*	28.20 ± 1.79	28.68 ± 2.10*

Results presented as mean ± SD; n = 5;* = there was no significant difference between Day-0 and Day-4 (P > 0.05); a = there was a significant difference between Day-0 and Day-4 (P < 0.05).

#### Effect of crude extracts of leaves of Croton macrostachyus and Acokanthera shimperi on parasitaemia and mean survival time of mice

Methanol and aqueous extracts of *Croton macrostachyus* and *Acokanthera shimperi* showed dose dependent chemosuppressive effect at various doses in mice infected with *Plasmodium berghei* parasite. The mice treated with chloroquine were completely free from the parasites on day four in all the experiments.

The crude methanol extracts of *Croton macrostachyus* significantly suppressed (P < 0.05) parasitaemia at all dose levels compared to the negative control groups but did not improve survival time significantly (P > 0.05) (Table 5).

**Table 5:** **Suppressive effect of methanol extracts of C** ***roton macrostachyus*** **and mean survival time**

Group	Dose (mg/kg)	Antimalarial activities on Day-4 post infection		Mean survival time in days
		% Parasitaemia ± SEM	% Suppression	
1	Distilled water (1 mL)	46.86 ± 1.03	0.00	7.20 ± 0.37
2	200	38.56 ± 3.07	21.06	7.80 ± 0.58^b^
3	400	33.88 ± 5.75	27.72	7.40 ± 0.98^b^
4	600	30.80 ± 5.56	34.33	7.40 ± 0.93^b^
5	Chloroquine (25)	0.00	100.00	11.75 ± 0.48*

% parasitaemia and mean survival time results presented as mean ± SD; n = 5; * = values are significantly different from that of the negative control; values with Superscript b- are not significantly different from that of the negative control (P > 0.05).

Similarly, the aqueous extracts of *Croton macrostachyus* significantly reduced parasitaemia compared to the negative control group. The highest significant parasitaemia suppression (P < 0.05) was observed for this extract although it did not improve the mean survival time (Table 6).

**Table 6:** **Suppressive effect of aqueous extract of** ***Croton macrostachyus*** **and mean survival time**

Group	Dose (mg/kg)	Antimalarial activities on Day-4 post infection		Mean survival time in days
		% Parasitaemia ± SEM	% Suppression	
1	Distilled water (1 mL)	46.89 ± 1.03	0.00	7.20 ± 0.20
2	200	34.24 ± 5.16	26.14	7.40 ± 0.98^b^
3	400	32.60 ± 4.50	30.50	7.80 ± 0.37^b^
4	600	23.20 ± 7.87	50.53	7.40 ± 0.60^b^
5	Chloroquine (25)	0.00	100.00	11.75 ± 0.48*

% parasitaemia and mean survival time results presented as mean ± SD; n = 5; * = values are significantly different from that of the negative control; values with Superscript b- are not significantly different from the negative control (P > 0.05).

Tables 7 and 8 show a summary of parasitaemia suppression (%) of *A. schimperi* for mice on day 4. Both aqueous and methanol extracts showed significant suppression of parasitaemia (P < 0.05). The mean survival time of treatment group ranged from 7.00 ± 1.73 up to 10.60 ± 0.51 days, whereas that of negative control was 6.2 ± 0.20 days. The mice treated with the methanol and aqueous extracts at 600 mg/kg survived longer (10.60 ± 0.51 days for methanol extract and 9.60 ± 0.51 day for aqueous extract) than those in the negative control group with mean survival time of 6.2 ± 0.20 days (P < 0.05).

**Table 7:** **Parasitaemia suppressive effect of methanol extract of** ***Acokanthera schimperi*** **and mean survival time**

Group	Dose (mg/kg)	Antimalarial activities on Day-4 post infection		Mean survival time in days
		% Parasitaemia ± SEM	% Suppression	
1	Distilled water (1 mL)	52.80 ± 4.24	0.00	6.2 ± 0.20
2	200	40.80 ± 3.67	22.7	7.00 ± 1.73^b^
3	400	36.00 ± 4.74	31.8	7.20 ± 1.3^b^
4	600	34.40 ± 2.87	34.8	10.60 ± 0.51*
5	Chloroquine (25)	0.00	100.0	14.2 ± 1.30*

% parasitaemia and mean survival time results presented as mean ± SD; n = 5; values with superscript b are not significantly different from that of the negative control (P > 0.05).*Values are significantly different as compared to that of negative controls (P < 0.05).

**Table 8:** **Parasitaemia suppressive effect of aqueous extract of** ***Acokanthera schimperi*** **and mean survival time**

Group	Dose (mg/kg)	Antimalarial activities on Day-4 post infection		Mean survival
		% Parasitaemia ± SEM	% Suppression	Time in days
1	Distilled Water (1 mL)	52.80 ± 4.24	0.00	6.20 ± 0.20
2	200	41.0 ± 3.89	22.34	6.40 ± 0.25^b^
3	400	40.80 ± 1.65	22.72	7.00 ± 0.77^b^
4	600	39.80 ± 3.49	24.60	9.60 ± 0.51*
5	Chloroquine (25)	0.00	100.00	14.20 ± 0.58*

% parasitaemia and mean survival time results presented as mean ± SD; n = 5; values with Superscript b are not significantly different from the negative control (P > 0.05).* = there was a significant difference compared to negative controls (P < 0.05).

## Discussion

In this study, the suppressive tests of extracts of the leaves of *Acokanthera schimperi* and *Croton macrostachyus* demonstrated a significant dose dependent chemosuppressive effect at various doses (200,400,600 mg/kg) for aqueous and methanol extracts. The highest percentage chemosuppression was exhibited by the aqueous extract of *Croton macrostachyus*, followed by the methanol extract of *A. schimperi* at 600 mg/kg.

Acute and sub-acute test results of the extracts of both plant species showed no sign of toxicity in all treated mice. The 4-day suppressive test is a standard test commonly used for antimalarial screening [24]. Extracts of *C. macrostachyus* and *A. schimperi* produced significant chemosuppression in all treated groups.

Antiplasmodial activity has been related to a range of several classes of secondary plant metabolites including alkaloids, sesquiterpenes, triterpenes, flavanoids, limonoids, quassinoids, xanthones, quinines and phenolic compounds of which alkaloids have been the most important and have shown very interesting activities [8, 29]. Indeed, quinine is the first antimalarial drug that belongs to the class of alkaloids [30] *Croton* spp. generally contain diterpenoids, triterpenoids, alkaloids, flavonoids, lignoids and proanthocyanidins [31], which have strong antiplasmodial activity. Therefore, the antiplasmodial activity observed in this study may be attributed to the presence of these bioactive compounds.

Both the methanol and aqueous extracts of *C. macrostachyus* exhibited comparable suppressive effect on *P. berghei*. However, aqueous extract of *Acokanthera schimperi* in all doses tested may be considered to have lower activity i.e. 22.34%, 22.72% and 24.60% parasitaemia reduction at 200, 400 and 600 mg/kg, respectively. On the other hand, methanol extract can be considered active with 34.8% parasitaemia reduction at 600 mg/kg and 31.8% at 400 mg/kg. Thus, the result of this study may justify the traditional use of the plant for antimalarial therapy in different parts of Ethiopia [13, 14].

Methanol and aqueous extract of *C. macrostachyus* and *A. schimperi* prevented body weight loss. Result of similar study on crude extracts and solvent fractions of *Croton macrostachyus* revealed the significant prevention of weight loss associated with increase in parasitemia level [32].

Hematological abnormalities are considered a hallmark of malaria [33]. According to Iyawe and Onigbinde [34], *Plasmodium berghei* increases erythrocyte fragility and significantly reduces PCV in mice. Methanol and aqueous extracts of *A. schimperi* prevented PCV reduction in a dose dependent manner as compared to negative control group.

Another parameter used to evaluate the efficacy of antimalarial plant extracts in this study was mean survival time. *A. schimperi* significantly prolonged mice survival time compared to negative control group at 600 mg/kg and this could be attributed to parasitemia suppression effect of the extract of the plant. In contrast, all the doses of *C. macrostachyus*, which showed higher parasitemia suppression in this study, did not prolong mean survival time. This may show that half-life of the active compounds in plasma metabolism is shorter [35].

Comparatively, the methanol extract of the leaves of *C. macrostachyus* showed lower antimalarial activity than its water extract counterpart. On the other hand, water extract of the leaves of *Acokanthera schimperi* showed lower activity compared to that of methanol. During extraction, solvents diffuse into the solid plant material and solubilize compounds with similar polarity [21]. This might indicate that the active compounds solubility which is responsible for the observed activity is different.

Chloroquine phosphate used in this study suppressed parasitaemia to non-detectable number, which is in agreement with [36] in which standard antimalarial drug cleared *P. b*ergh*ei*, to undetectable level. The results of the acute toxicity within 24 hours revealed that no toxic effect or mortality was observed in mice treated orally with methanol and aqueous extracts of *Croton macrostachyus* and *Acokanthera schimperi* as a single dose of 2000 mg/kg, which the single highest dose is recommended by OECD Guidelines 425 for testing acute toxicity. In addition, no changes in general appearance or behavioral pattern were noted until the end of 14 days. Therefore, absence of mortality up to an oral dose of 2000 mg/kg could indicate that the test extracts are safe and this could in turn explain the safe use of the plants by local people in Ethiopia who have been using them in traditional management of malaria [13, 14].

The median lethal oral dose (LD_50_) is greater than 2000 mg/kg if three or more animals survive [23]. No death was observed in the animals receiving the extracts up to a dose of 2000 mg/kg body weight, which is about 10 times the minimum effective dose tested (200 mg/kg). If a test substance has LD_50_ higher than three times the minimum effective dose, it can be taken as a good candidate for further studies [26]. The extracts from this study are, therefore, good candidates for further studies.

Repeated dose toxicity studies provide information on the possible health hazards likely to arise from repeated exposure over a relatively limited period of time [37] and repeated daily dosing is more clinically relevant than acute toxicity study. In this study, a 4-day administration of the extracts of 500, 750 and 1000 mg/kg daily revealed no sign of toxicity and all mice survived beyond the 30-days observation period.

According to Pillai *et al*. [38], the reduction in body weight is a simple and sensitive index of toxicity after exposure to toxic substances. In this study, there was no significant body weight change for aqueous and methanol extracts of *A. schimperi* and methanol extract of *C. macrostachyus*. Nevertheless, body weight loss in mice was observed at 1000 mg/kg in those treated with aqueous extract of *C. macrostachyus*. The decrease in body weight observed in the group treated with the highest dose could be attributed to the suppression of the animals’ appetite by the extract.

Investigation of the hematological parameters can also be used to determine the extent of deleterious effect of foreign compound including plant extracts on the blood constituent of an animal. The hematological parameters of treated mice (Hb and PCV of *A. schimperi* and PCV of *C. macrostachyus*), an index of anemia, did not show significant difference on day 4 compared to day 0. Similarly, WBC, in case of *C. macrostachyus* treated mice, which is an important index of pathological and physiological status [28], exhibited no significant difference. This effect of the extract on the hematological parameters of the animals might be an indication that it is unlikely to be toxic.

## Conclusions

The antimalarial activity test showed that both plants exhibited significant antiplasmodial activity as evidenced by their ability to suppress *P. berghei* infection in mice in a dose dependent manner, which may partly justify the claim by traditional practitioners about the use of these two plants against malaria. The study also revealed that the two plants are not toxic in both acute and sub-acute tests at the tested doses of the extracts. Further evaluation of these plants is, however, needed to isolate, identify and characterize the active ingredients responsible for the observed antimalarial activity of the plants.
